# Lipoprotein-associated phospholipase A_2 _(Lp-PLA_2_) activity, platelet-activating factor acetylhydrolase (PAF-AH) in leukocytes and body composition in healthy adults

**DOI:** 10.1186/1476-511X-8-19

**Published:** 2009-06-05

**Authors:** Paraskevi Detopoulou, Tzortzis Nomikos, Elizabeth Fragopoulou, Demosthenis B Panagiotakos, Christos Pitsavos, Christodoulos Stefanadis, Smaragdi Antonopoulou

**Affiliations:** 1 Department of Nutrition-Dietetics, Harokopio University, Athens, Greece; 2 First Cardiology Clinic, School of Medicine, University of Athens, Athens, Greece

## Abstract

**Background:**

Lipoprotein-associated phospholipase A_2 _(Lp-PLA_2_) also known as serum platelet activating factor acetylhydrolase (PAF-AH) activity constitutes a novel risk marker for cardiovascular disease. Leukocytes constitute one main cellular source of circulating Lp-PLA_2_. The aim of the present study was to evaluate the association of both serum and leukocyte PAF-AH activities with fat distribution and lean tissue. One hundred healthy volunteers without cardiovascular disease history participated in this study (n = 52 men, 44 ± 13 years and n = 48 women, 43 ± 13 years). Body composition was assessed with dual-energy X-ray absorptiometry, while anthropometrical indices were also measured. The activity of Lp-PLA_2 _and levels of lipid and glycemic parameters were determined in fasting samples.

**Results:**

Mean Lp-PLA_2 _activity was 24.8 ± 4.5 and 19.6 ± 5.0 nmol/min/mL in men and women, respectively (P < 0.001). Mean activity of PAF-AH in leukocyte homogenates was 386 ± 127 pmol/min/mg and 292 ± 92 pmol/min/mg in men and women, correspondingly (P < 0.001). In multiple regression models upper and total adiposity measures were positively associated with Lp-PLA_2 _activity in men after adjusting for LDL-cholesterol, age, smoking, hs-CRP and physical activity, whereas no associations were found with PAF-AH leukocyte homogenates activity. Hierarchical analysis revealed that the variables with the highest explanatory ability of Lp-PLA_2 _activity in men, were DXA deriving L1–L4 region of interest and arms fat (increase in R^2 ^= 0.136, P = 0.005 and increase in R^2 ^= 0.118, P = 0.009, respectively), followed by trunk fat and total fat. In women, no association of body composition variables with Lp-PLA_2 _nor PAF-AH leukocyte homogenates activity was found.

**Conclusion:**

Lp-PLA_2 _activity is differentiated across levels of adiposity and topology of adipose tissue, whereas no association was found regarding PAF-AH leukocyte homogenates activity. Our findings suggest that Lp-PLA_2 _may compensate for the adiposity-associated increases in inflammatory and oxidative burden, in men.

## Background

Adipose tissue has been well recognised as an endocrine organ as it secretes several molecules with endocrine, paracrine and autocrine actions. Moreover, its accumulation at the upper body compartments results in the infiltration of macrophages [1] and the concomitant production of pro-inflammatory, pro-oxidant and pro-thrombotic factors, such as tumor-necrosis factor alpha (TNF-α), interleukin-6 (IL-6), platelet activating factor (PAF), PAF-like molecules and oxidized phospholipids [2-4]. This local inflammatory burst characterized with tissue remodelling, macrophage activation and enhanced chemoattraction of leukocytes together with the predominance of a systemic low grade inflammatory state turn the adipose tissue into a key player in atherosclerosis progress and inflammation [5]. On the other hand, lean tissue has been found to be inversely related to inflammatory indices [6].

Lipoprotein associated phospholipase A_2 _(Lp-PLA_2_), also known as plasma platelet activating factor acetylhydrolase (PAF-AH), is a Ca^2+^-independent enzyme implicated in inflammation and atherosclerosis [7]. It is mostly bound to LDL particles and in a lesser extent to HDL and VLDL lipoproteins and it is mainly produced by monocytes, macrophages, platelets and mast cells [7]. An accumulating body of evidence supports that Lp-PLA_2 _is a novel risk marker for cardiovascular disease [8]. As far as its action is concerned, it cleaves short chain acyl chains at the *sn*-2 position of phospholipids [7] such as, oxidized phospholipids and PAF [9], which is strongly implicated in atherosclerosis and inflammation [10]. From this point of view, Lp-PLA_2 _could be characterized as an anti-atherogenic enzyme. However, its action goes hand in hand with the production of inflammatory molecules, such as lyso-phosphatidylcholine and oxidized non esterified fatty acids [7], which are proatherogenic.

According to recent observations, adiposity may be implicated in the regulation of Lp-PLA_2 _activity together with age, gender and LDL-cholesterol [11,12], although not all studies agree [3]. This field has become even more complicated since epidemiological studies are inconsistent as far as the relation of body mass index (BMI) with Lp-PLA_2_is concerned [12-19]. No study, so far, has investigated the association of both central and peripheral fat, as well as lean tissue with Lp-PLA_2_, despite the urgent need for an in-depth analysis of Lp-PLA_2 _determinants. The present work aimed at evaluating the relation of Lp-PLA_2 _activity in serum and the enzyme activity in leukocytes (PAF-AH in leukocytes), a major cellular source of the circulating enzyme, with body composition as assessed with dual X-ray absorptiometry (DXA) in healthy adults.

## Methods

### Subjects

Fifty two (52) men and forty eight (48) age- and BMI-matched women (15 menopausal) from the Athens greater area, were studied in our Institution. Exclusion criteria were medical treatment, history of cardiovascular or any other inflammatory disease, cold or flu, acute respiratory infection, dental problems and renal/hepatic abnormalities.

### Bioethics

The protocol was approved by the Bioethics Committee of Harokopio University and was in accordance with the Declaration of Helsinki (1989) of the World Medical Association and all participants gave their informed written consent in order to participate in the study.

### Lifestyle variables assessment

Smokers were classified as current smokers (smoking daily at least one cigarette per day), former smokers and never smokers. Physical activity was assessed with the use of a validated questionnaire (IPAQ-short version) and was expressed in metabolic equivalents minutes per week (MET-min-week) [20].

### Anthropometric measurements

Weight and height were measured in light clothing and without shoes. BMI was then calculated as weight (kg) divided by height^2 ^(m^2^). Waist circumference was measured between the superior iliac crest and the lower rib margin in the midaxillary line and hip circumference as the maximal horizontal circumference at the level of the buttocks. Both circumferences were measured to the nearest 0.1 cm and waist to hip ratio was calculated. Sagittal diameter was measured with an anthropometer (Lafayette Instr.) as previously suggested [21]. The abdominal skinfold was measured 3 cm lateral and 1 cm inferior to the midpoint of the umbilicus with a Harpenden caliper (Harpenden, UK) in triplicate to the nearest 0.1 mm. All measures were performed at the right side of the body by the same individual.

### DXA measurements

Body composition was assessed by DXA (Lunar, Corporation, Brussels, Belgium) set at medium speed and according to the manufacturer's instructions. Prior to the scanning procedure, subjects were asked to remove all materials that could attenuate the X-ray beam. Besides the standard body composition analysis (software version 4.6), for each subject a "region of interest" (ROI) was manually defined as a quadrilateral box around the L1–L4 area as previously described [22]. The fat in this ROI is strongly correlated with visceral adipose tissue as determined from CT scans (gold standard method) [22] and is a more informative measure of central adiposity than trunk fat in women especially, given that trunk fat in females includes the sex-specific (i.e. breast) fat. To eliminate the between examiners errors all the analysis were performed by the same individual.

### Isolation of leukocytes from heparinized blood

Five mL of heparinized blood were obtained from each volunteer. In order to induce erythrocyte sedimentation, 1.7 mL of dextran solution (3% dextran in NaCl 0.15 M) was added and the mixture was kept for 1 h at room temperature. The leukocyte rich supernatant was then centrifuged at 500 × g for 10 min at room temperature. Contaminating erythrocytes of the sediment were lysed with the addition of a lysis solution consisting of 155 mM NH_4_Cl, 10 mM KHCO_3 _and 0.1 mM EDTA and then removed with a centrifugation at 300 × g for 10 min at room temperature. The pelleted cells were resuspended in 1 mL of a buffer containing 50 mM Tris-HCl (pH 7.4), 0.25 M sucrose and 1 mM DTT and then sonicated on ice for 4 times of 10 sec each. The leukocyte homogenate was aliquoted and stored at -80°C. Protein concentrations of all preparations, were determined according to the Bradford method [23] with the use of BSA as protein standard.

### Biochemical measurements

PAF-AH and Lp-PLA_2 _activities in leukocyte homogenates and serum respectively were determined by the trichloroacetic acid precipitation method using [^3^H] PAF as a substrate. Briefly, leukocyte homogenates (containing 60 μg of total protein) or 2 μL of serum were incubated with 4 nmol of [^3^H] PAF (20 Bq per nmol) for 15 min, at 37°C in a final volume of 200 μL of 100 mM Tris/HCl buffer (pH 7.2) containing 1 mM EGTA. The reaction was terminated by the addition of cold trichloroacetic acid (10% final concentration). The samples were then placed in an ice bath for 30 min and subsequently centrifuged for 2 min at 14000 g. The [^3^H]-acetate released into the aqueous phase was measured on a liquid scintillation counter. All assays were performed in duplicate. The enzyme activity was expressed as nmol of PAF degraded per min per mg of leukocyte homogenate protein or per mL of serum.

Serum glucose, triacylglycerols, total cholesterol and HDL cholesterol were determined enzymatically in fasting samples (ACE analyzer, Schiapparelli Biosystems, Inc, New Jersey, USA) using reagents from Alfa Wassermann (Woerden, The Netherlands). LDL was calculated with the Friedewald Formula. High sensitivity C-reactive protein was measured in a LISA 200 analyser (Biocode, Hycel) using commercially available reagents (Siemens Healthcare Diagnostics).

### Statistical analysis

Normally distributed continuous variables are presented as mean values ± standard deviation, while skewed variables as median and quartiles. Categorical variables are presented as relative frequencies (%). For the comparisons between genders the independent samples t-test for normally distributed variables or the Mann-Whitney of the skewed and the chi-square test for categorical variables, were used. Correlations were evaluated using the Pearson coefficient for the normally distributed and the Spearman for the skewed variables. Regression models were applied in order to evaluate the effect of adiposity measures (as explanatory variables) on Lp-PLA_2 _activity or PAF-AH activity in leukocytes (as dependent outcomes), after adjustment for LDL-cholesterol, age, smoking and physical activity (covariates), which are known to determine Lp-PLA_2 _activity [12-15,24]. Moreover, smoking and physical activity were also included in the models as they are related to Lp-PLA_2 _activity through the oxidative modification of LDL-particles [25]. In order to test whether the relations of Lp-PLA_2 _activity with body fat compartments are influenced by inflammatory state, C-reactive protein was also entered in the regression models. Normality of the residuals of the regression analyses was tested through P-P plots and the Kolmogorov-Smirnoff criterion, while the assumptions of non co-linearity, homoscedacity and independency were tested using the scatter plots of predicted against standardised residuals and the calculation of the Variance Inflation Factor. Results from regression models are presented as b-coefficients and standard error. In order to "hierarchy" the explanatory ability of adiposity variables on Lp-PLA_2 _activity, additive regression models were applied with age, LDL-cholesterol, smoking and physical activity as the core model and each adiposity measure as the additive variable. The change in R-squared values (i.e. the coefficient of determination, which represents the proportion of variability in the data that is accounted for by the regression model) was calculated and the corresponding F-test assisted in order to evaluate significance.

We have also assessed the relation of Lp-PLA_2 _activity and PAF-AH in leukocytes with patterns of adipose and lean tissue measures with a multivariate technique, the Principal Component Analysis (PCA). In this way, after evaluating the correlations between continuous variables, new variables were created which "summarize" the existing information. To decide the number of components to retain from the PCA, the eigenvalues that derived from the correlation matrix of the standardized variables were examined (the eigenvalue evaluates the proportion of the variance in consumption explained by each extracted component). According to the Kaiser criterion, the number of components that should be retained is equal to the number of eigenvalues that are greater than one. Based on the principle that the component scores are interpreted similarly to correlation coefficients (thus, higher absolute values indicate that the body composition variable contributes most to the formulation of a component), the body composition patterns were defined in relation to scores of the body composition variables that correlated most with the factor (scores >0.5 were used). The orthogonal rotation with varimax option was used to derive optimal, non-correlated body composition components (i.e. body composition patterns). The information was rotated in order to increase the representation of each body composition to a component. All reported P-values were based on two-sided tests and compared to a significance level of 5%. SPSS 14 (SPSS Inc., Chicago, Illinois, USA) software was used for statistical analysis.

### Results

Mean Lp-PLA_2 _activity was 24.8 ± 4.5 nmol/min/mL in men and 19.6 ± 5.0 nmol/min/mL in women (P < 0.001) (range 15.7–37.8 and 11.0–37.1 nmol/min/mL in men and women, respectively). Mean activity of PAF-AH in leukocyte homogenates was 386 ± 127 pmol/min/mg in men (ranging from 142 to 714 pmol/min/mg) and 292 ± 92 pmol/min/mg in women (ranging from 96 to 545 pmol/min/mg), correspondingly (P < 0.001). Table 1 demonstrates the basic anthropometric and clinical characteristics of participants. There was no significant difference between men and women regarding age, BMI and smoking habits, whereas differences in certain biochemical parameters and body composition measurements, such as total body fat and central adiposity were observed. Moreover, positive correlations were documented between Lp-PLA_2_activity and total- (r = 0.588, P < 0.001 in men and r = 0.645, P < 0.001 in women), LDL-cholesterol (r = 0.531, P < 0.001 in men and r = 0.574 and P < 0.001 in women) and triglycerides (log-values) (r = 0.504, P < 0.001 in men and r = 0.352, P = 0.012 in women) (data not shown). PAF-AH in leukocyte homogenates was associated with LDL-cholesterol and Lp-PLA_2 _in women (r = 0.338, p = 0.013 and r = 0.278, p = 0.049), whereas in men no association was found between PAF-AH in leukocyte homogenates with lipid parameters or Lp-PLA_2 _activity (data not shown). Interestingly, PAF-AH in leukocyte homogenates was associated with CRP levels (r = 0.338, p = 0.023 and r = 0.316, p = 0.019 in men and women, respectively) whereas the activity of Lp-PLA_2 _showed no similar trend (data not shown).

**Table 1:** Selected biochemical and anthropometric characteristics of the subjects.

	**Men ***(n = 52)*		**Women ***(n = 48)*		**P**
	**Mean or Median†**	**SD or quartiles**	**Mean or Median**	**SD or quartiles**	

Age (years)	43.7	13.3	42.8	12.8	0.726^a^
Body mass Index (kg/m^2^)	27.4	3.8	25.7	5.6	0.087^a^
Current smokers (%)	34.7		27.6		0.640^b^
MET (minutes per week)	1043	1046	951	954	0.658^a^
Lp-PLA_2 _activity (nmol/min/mL)	24.8	4.5	19.6	5.0	<0.05^a^
PAF-AH activity in leukocyte homogenates (pmol/min/mg)	386	127	292	92	<0.05^a^
Glucose (mmol/L)	5.22	0.43	5.02	0.72	0.101^a^
Triglycerides (mmol/L)	1.34	0.89–1.71	0.75	0.56–1.04	<0.05^a^
Total cholesterol (mmol/L)	5.66	0.97	5.47	1.20	0.396^a^
HDL-cholesterol (mmol/L)	1.10	0.20	1.28	0.31	<0.05^a^
LDL-cholestrerol (mmol/L)	3.89	0.78	3.74	1.02	0.401^a^
C-reactive protein (mg/dL)	0.40	0.34–0.49	0.39	0.31–0.48	0.438^a^
Waist circumference (cm)	92.8	9.2	77.7	16.1	<0.05^a^
Hip circumference (cm)	104.7	7.0	103.8	15.7	0.696^a^
Waist- to- hip ratio	0.9	0.1	0.7	0.1	<0.05^a^
Sagittal diameter (cm)	23.4	3.4	20.1	4.5	<0.05^a^
Abdominal skinfold (mm)	28.3	7.2	27.4	10.0	0.612^a^
% Total body fat	26.7	5.2	37.2	8.4	<0.05^a^
DXA ROI fat (kg)	3.0	0.9	2.7	1.5	0.116^a^
Legs fat (kg)	6.8	3.0	9.2	3.9	<0.05^a^
Arms lean tissue (kg)	3.81	0.60	6.67	1.18	<0.05^a^
Legs lean tissue (kg)	12.45	1.95	18.78	2.85	<0.05^a^

MET: Metabolic equivalent, ROI: Region of interest, DXA: dual energy X-ray absorptiometry

† Data are presented as mean ± standard deviation for normally distributed variables. Otherwise data are presented as median ± lower (Q_0.25_) and upper (Q_0.75_) quartile.

^a ^Student t-test was used to compare means.

^b ^Chi-square test was used to compare percentages in the two groups.

In Table 2 the unadjusted Pearson correlation coefficients between Lp-PLA_2 _activity, PAF-AH in leukocyte homogenates and various body composition variables are displayed. Data are presented separately in men and women since a significant interaction was observed between anthropometry or body composition variables and gender on the investigated outcome (P < 0.05). Associations of adiposity parameters with Lp-PLA_2 _and PAF-AH in leukocyte homogenates were more prominent in men. Particularly, Lp-PLA_2 _activity was positively associated with trunk fat, DXA ROI fat, arms fat and total fat and marginally associated with waist circumference (P = 0.10) in men, whereas in women no association was detected between the enzyme activity and body fat compartments. PAF-AH in leukocytes homogenates displayed a similar pattern, since it was positively associated with DXA ROI fat and % total body fat in men, whereas in women no association with body fat indices was observed. Legs lean mass was inversely related to the activity of both Lp-PLA_2 _and PAF-AH in leukocytes activities in women, whereas an inverse association of arms lean mass with PAF-AH activity in leukocytes was observed in men.

**Table 2:** Pearson correlation coefficients of Lp-PLA_2 _and leukocyte PAF-AH activities with body composition indices in men and women.

	Serum Lp-PLA_2 _activity		PAF-AH activity in leukocytes homogenate	
	Men *(n = 52)*	Women *(n = 48)*	Men *(n = 52)*	Women *(n = 48)*

Body mass index (BMI) (kg/m^2^)	0.206 (P = 0.170)	-0.051 (P = 0.718)	0.163 (P = 0.284)	-0.044 (P = 0.746)
Waist circumference (cm)	0.240 (P = 0.100)	-0.005 (P = 0.975)	0.146 (P = 0.327)	-0.218 (P = 0.114)
Hip Circumference (cm)	0.202 (P = 0.168)	-0.043 (P = 0.766)	0.070 (P = 0.639)	-0.037 (P = 0.791)
Sagittal diameter (cm)	0.173 (P = 0.252)	0.007 (P = 0.963)	0.178 (P = 0.243)	0.088 (P = 0.532)
Abdominal skinfold (mm)	0.170 (P = 0.253)	0.086 (P = 0.554)	0.022 (P = 0.883)	0.087 (P = 0.531)
DXA ROI fat (kg)	0.380 (P = 0.008)	-0.088 (P = 0.535)	0.247 (P = 0.094)	-0.020 (P = 0.883)
Trunk fat (kg)	0.356 (P = 0.013)	-0.063 (P = 0.663)	0.162 (P = 0.277)	-0.040 (P = 0.772)
Arms fat (kg)	0.318 (P = 0.028)	0.083 (P = 0.563)	0.097 (P = 0.518)	-0.186 (P = 0.174)
Legs fat (kg)	0.084 (P = 0.572)	-0.099 (P = 0.488)	0.149 (P = 0.316)	-0.132 (P = 0.338)
% total body fat	0.229 (P = 0.118)	0.006 (P = 0.967)	0.296 (P = 0.044)	-0.071 (P = 0.608)
Total body fat (kg)	0.253 (P = 0.083)	-0.064 (P = 0.657)	0.167 (P = 0.263)	-0.098 (P = 0.475)
Arms lean tissue (kg)	0.004 (P = 0.979)	-0.093 (P = 0.518)	-0.333 (P = 0.022)	-0.152 (P = 0.266)
Legs lean tissue (kg)	0.058 (P = 0.698)	-0.318 (P = 0.023)	-0.222 (P = 0.133)	-0.219 (P = 0.100)

P values are shown in parenthesis. All variables were normally distributed according to Kolmogorov Smirnoff Kriterion.

In Table 3 the associations of anthropometry and body composition variables with Lp-PLA_2 _activity are displayed, after adjusting for LDL-cholesterol, age, smoking, hs-CRP and physical activity. Fat in the DXA ROI, arms fat, trunk fat and total body fat were positively associated with Lp-PLA_2 _activity in men, while no associations were found in women. Lean tissue in arms and legs were also included in similar regression models but were not significant predictors of Lp-PLA_2 _activity. A *posterior *observed power for these non significant associations in women was about 45%. For this reason more emphasis will be given to the observed relations in men throughout this work. The associations of PAF-AH activity in leukocytes with body fat and lean tissue indices were not significant in age adjusted multiple regression models and fully adjusted multiple regression models (data not shown), in which PAF-AH activity in leukocyte homogenates was used as a dependent variable.

**Table 3:** Multi-adjusted linear regression models with Lp-PLA_2 _as dependent variable.

	Men *(n = 52)*			Women *(n = 48)*		
	B	SE	*P*	B	SE	*P*

***Model 1***						
LDL-cholesterol (mmol/L)	3.140	0.869	0.001	4.084	0.632	<0.001
Age (years)	-0.077	0.053	0.156	-0.078	0.051	0.137
Smoking (never/former vs. current smoker)	-0.570	1.324	0.669	1.067	1.145	0.357
MET minutes per week (× 100)	0.002	0.001	0.768	0.000	0.001	0.640
hs C-reactive protein (mg/dL)	3.891	2.623	0.147	-1.166	2.470	0.640
***Model 1 + one variable of the following:***						
*Body mass index (kg/m^2^)*	0.227	0.161	0.168	-0.021	0.121	0.860
*Waist circumference (cm)*	0.097	0.068	0.160	0.022	0.038	0.565
*Hip circumference (cm)*	0.161	0.082	0.057	0.019	0.034	0.579
*Waist-to-hip ratio*	1.753	11.422	0.879	1.084	4.158	0.796
*Sagittal diameter (cm)*	0.267	0.197	0.184	-0.039	0.139	0.784
*Abdominal skinfold (mm)*	0.132	0.092	0.161	0.022	0.061	0.720
*DXA ROI fat (kg)*	1.867	0.620	0.005	-0.270	0.447	0.549
*Trunk fat (kg)*	0.409	0.151	0.010	-0.068	0.111	0.546
*Arms fat (kg)*	2.313	0.835	0.009	0.195	0.568	0.734
*Legs fat (kg)*	0.299	0.197	0.138	-0.042	0.447	0.776
*% total body fat*	0.218	0.106	0.046	-0.045	0.076	0.647
*Total body fat (kg)*	0.178	0.078	0.029	-0.023	0.557	0.690

MET: Metabolic equivalent; ROI: region of interest; DXA: dual energy X-ray absorptiometry.

All models are also adjusted for LDL-cholesterol, age, smoking and physical activity.

Table 4 presents *R*-squared values, the corresponding F-tests and p-values in various additive models that evaluated the explanatory ability of body composition parameters on Lp-PLA_2 _activity. It is noteworthy that LDL-cholesterol, age, smoking and physical activity when regarded as a variables block explained approximately 30% of the variability of the enzymatic activity, which is in line with the results of other studies [12]. Although there were no significant differences between consecutive R^2 ^values, the hierarchy of anthropometric indices is of importance in clinical settings. The variable with the highest significant additive explanatory ability on top of background confounding was fat in the DXA ROI (i.e. R^2 ^change = 0.136, P = 0.005), followed by arms fat, trunk fat and total body fat (Table 4).

**Table 4:** R-squared values, and corresponding F-tests in various models that evaluated the explanatory ability of body composition parameters towards Lp-PLA_2 _activity.

**Initial model:**	**R^2^**	**F**	***P***
LDL-cholesterol, age, smoking, physical activity	0.296	4.098	0.007

**Additive models **(initial + one of the following variables):	R^2 ^change*	**F**	***P***
*DXA ROI fat*	0.136	9.076	0.005
*Arms fat*	0.118	7.678	0.009
*Trunk fat*	0.114	7.316	0.010
*Total body fat*	0.084	5.152	0.029

ROI: Region of interest; DXA: dual energy X-ray absorptiometry.

The other anthropometric variables tested did not have a significant additive explanatory effect on Lp-PLA_2 _activity.

* The change in R-squared values between the additive and the initial models.

Given that lean tissue has been found to be inversely related to inflammatory markers and strong correlations exist between adiposity and lean tissue measures, we evaluated the relation of Lp-PLA_2 _activity and PAF-AH in leukocytes with patterns of adipose and lean tissue measures with the technique of PCA. In the PCA analysis two components of body composition were extracted in men and women, i.e. the "fatness" (that was loaded by total body fat, DXA ROI fat, arms fat and legs fat) and the "leanness" (total lean tissue, legs lean tissue and arms lean tissue) (Table 5). These components were then entered in the multiple adjusted regression models, in order to evaluate the effects of fat and lean tissue on Lp-PLA_2 _activity and PAF-AH activity in leukocyte homogenates. In men, the fatness component was a significant predictor of Lp-PLA_2 _activity in multi-adjusted analysis, whereas in age adjusted regression models it was not a significant predictor of PAF-AH activity in leukocyte homogenates. In women, neither the fatness component nor the leanness component showed any association with the measured enzymatic activities (Table 6).

**Table 5:** Score coefficients derived from principal components analysis regarding body composition variables and identification of body composition patterns for male and female participants.

	*Men*		*Women*	
	Component 1: Fatness	Component 2: Leanness	Component 1: Fatness	Component 2: Leanness

*DXA ROI fat*	**0.915**	-0.142	**0.937**	0.118
*Arms fat*	**0.948**	0.093	**0.946**	0.152
*Legs fat*	**0.897**	0.208	**0.909**	0.290
*Total fat*	**0.990**	0.088	**0.968**	0.217
*Arms lean*	-0.095	**0.946**	0168	**0.893**
*Legs lean*	0.230	**0.926**	0.187	**0.902**

Score coefficients are similar to the correlation coefficients. Higher absolute values indicate that the body composition variable is correlated with the respective component. Numbers in bold indicate loadings greater than 0.5.

**Table 6:** Multiple linear regression models with Lp-PLA_2 _or PAF-AH in leukocytes homogenates as dependent variable and PCA component scores as independent variables.

	**†Lp-PLA_2_**				**‡PAF-AH in leukocyte homogenates**			
	**Men *(n = 52)***		**Women *(n = 48)***		**Men *(n = 52)***		**Women *(n = 48)***	

	b ± SE	P	b ± SE	P	b ± SE	P	b ± SE	P

*"Fatness" scores*	1.43 ± 0.58	0.019	-0.40 ± 0.66	0.548	25.98 ± 20.31	0.209	4.61 ± 16.42	0.780

*"Leaness" scores*	0.05 ± 0.75	0.946	0.24 ± 0.56	0.673	-35.62 ± 28.55	0.193	-21.06 ± 13.29	0.120

† All models were adjusted for age, smoking habits, physical activity (METs per week) and LDL-cholesterol

‡ All models were adjusted for age, smoking habits and physical activity (METs per week).

## Discussion

This is the first study evaluating the relation of Lp-PLA_2 _activity and PAF-AH activity in leukocytes to fat distribution in healthy adults. Main findings indicate that measures of upper adiposity (i.e. fat in the DXA ROI and arms fat) had the highest explanatory ability of Lp-PLA_2 _activity among other indices of adiposity in men in multi-adjusted models, whereas adiposity was not a significant determinant of PAF-AH activity in leukocyte homogenates.

Our results should be viewed taking into consideration existing studies, in which BMI and/or classic anthropometric indices were measured. In particular, weight loss has been found to induce reductions in Lp-PLA_2_activity in obese women [11]. Patients with the metabolic syndrome, which are characterized with central adiposity display higher levels of Lp-PLA_2 _[26,27], while Lp-PLA_2 _concentration has been positively correlated with measures of central adiposity in obese children [28] and in adults [29]. Epidemiological studies tend to show no relation of adiposity to Lp-PLA_2 _with some exceptions [12-19]. These discrepancies could be attributed to the inherent weaknesses of BMI as an index of adiposity [30] as it does not reflect fat distribution nor distinguishes between lean and fat mass.

In this context, the DXA-based body composition analysis performed in our study is informative as far as adipose and lean tissue compartmentalization is concerned. The magnitude of the additive explanatory ability of arms and trunk adiposity for Lp-PLA_2 _activity was approximately 10% after adjustments for the effects of classic confounders, such as LDL-cholesterol, smoking, hs-CRP levels, age and physical activity. This contradicts the generally believed view that Lp-PLA_2 _is minimally correlated with risk factors other than age, gender and LDL-cholesterol [13]. We believe that in male participants arm adiposity represents an upper fat distribution and reflects the effects of trunk adiposity on Lp-PLA_2_. This was also shown from the PCA analysis, in which subjects displaying the "fatness pattern" had increased values for all body fat compartments. Leg fat was not shown to have any protective effect against Lp-PLA_2 _possibly because of the fact that male participants accumulated fat mainly in the central area. This relation of Lp-PLA_2 _to upper fat depots can be better understood in the light of visceral adipose tissue metabolic effects given that DXA L1–L4 ROI fat and trunk adiposity are strongly correlated with visceral adipose tissue as determined from CT scans [22]. Indeed, lipolytic stimuli of visceral fat can lead to increases in free fatty acids which are connected to oxidative stress [31] and possibly implicated along with Lp-PLA_2 _in the atherosclerosis process [32]. Moreover, visceral fat has been positively related to small dense LDL particles, in which Lp-PLA_2 _is preferentially bound [33]. It is noteworthy that in a recent publication from the Framingham Heart Study no correlation of Lp-PLA_2 _activity nor mass was detected with visceral fat [3], whereas preliminary results from the HERITAGE study indicated a positive association of Lp-PLA_2 _activity with visceral fat [34]. For the interpretation of the above conflicting results, it should be noted that the Framingham Heart study participants were old (mean age 60 years) and medically treated (30% of the participants were chronic aspirin users) [3], whereas in the present study the participants were much younger, healthy and not medically treated.

The relation of adiposity to Lp-PLA_2 _observed in our study may be explained through the pleiotropic molecules being secreted by adipocytes, such as TNF-α, which may induce Lp-PLA_2 _secretion from various cells [35]. In addition, we should notice that an increase in Lp-PLA_2 _can also be mediated by an increase in PAF and oxidized phospholipids levels, resulting from the adipose tissue inflammatory burst, in order to degrade these bioactive molecules. The inclusion of hs-CRP in regression models did not attenuate the magnitude of the documented relationship between Lp-PLA_2 _activity and adiposity indices. This finding is in line with evidence suggesting that Lp-PLA_2 _is not associated in the present and other studies with CRP [12,13,36], although some studies report an association of CRP and Lp-PLA_2 _[15], and lends evidence to the fact that that more specific mediators of inflammation (e.g. TNF-α) may potentiate the increases in Lp-PLA_2 _in cases of fat accumulation. Moreover, adipocytes can also secrete PAF and have detectable amounts of PAF-AH [4,37] and may thus contribute in circulating levels of Lp-PLA_2_, although its main cellular source are macrophages [7]. Adipose-tissue deriving hormones, such as leptin, which is increased in obesity, may provide an additional mechanism through which adipose tissue may influence Lp-PLA_2_activity [18]. A leptin mediated upregulation of Lp-PLA_2_, however, most probably does not explain the relations documented in the present study, given that leptin was not correlated with Lp-PLA_2 _in men (r = 0.189, P = 0.309) (data not presented).

As far as PAF-AH activity in leukocyte homogenates is concerned, it was positively correlated with measures of adiposity in males and negatively associated with lean tissue in males and females but these associations were not significant in age- and multi-adjusted models. The differentiated nature of adiposity related regulation of PAF-AH activity in leukocyte homogenates and Lp-PLA_2 _activity in the volunteers studied may be explained by the inherent differences of the enzymatic activities measured. Firstly, although leukocytes constitute a source of Lp-PLA_2 _activity other cells can also produce and secrete Lp-PLA_2_, such as platelets, adipocytes, macrophages resident in adipose tissue etc. [7,37]. Indeed, in the present study platelet count was significantly correlated with Lp-PLA_2 _activity in men (r = 0.318, p = 0.031). Moreover, the production of Lp-PLA_2 _from leukocytes may be higher in pathological conditions, in which these cells are stimulated [35] compared to a relatively steady state in healthy volunteers included in the present study. Secondly, leukocytes contain not only the serum type Lp-PLA_2 _but also the intracellular type I PAF-AH [38], which are both measured in the assay used. However, the relative contribution of the type I PAF-AH in the activity measured is negligible [38]. Thirdly, the possible differences in the regulation of enzymatic activities measured are reflected to the different patterns of associations with inflammatory indices documented in this and other studies [36].

Limitations of our study include its cross-sectional nature, which can only generate hypothesis and not provide causal explanations. Lack of visceral fat mass data (by CT or MRI) is another limitation in the current study. However, it is probable that the observed relations between upper fat depots and Lp-PLA_2 _reflect the effects of visceral compartments since no relation was detected with central measures of subcutaneous fat (i.e. abdominal skinfold thickness) and Lp-PLA_2_. Moreover, the presented results for women concern both pre- and post- menopausal women. Separate analysis was difficult given the small number of female volunteers in each subgroup. Last but not least, it should be noted that in the present study total Lp-PLA_2 _activity was measured although LDL- and HDL-associated Lp-PLA_2 _activity determinations could be more informative in the light of the apparently different actions of Lp-PLA_2 _attached to these subfractions. However, the given results would not be significantly differentiated if HDL-associated activity was included, since as Tellis et al discuss the HDL-associated Lp-PLA2 activity represents about 5% of the total plasma enzyme activity, and does not considerably contribute to the total plasma enzyme activity [7].

## Conclusion

In conclusion, this is the first study to substantiate a differentiation in Lp-PLA_2 _activity across upper adiposity levels in healthy men, whereas PAF-AH activity in leukocytes was not related to measures of adiposity nor lean tissue in the subjects studied. Our innovative findings suggest that Lp-PLA_2 _activity may increase in order to compensate for the adiposity-associated increases in inflammatory and oxidative burden.

## Competing interests

The authors declare that they have no competing interests.

## Authors' contributions

PD wrote the manuscript, interpreted the results and designed the study, TN designed the study, interpreted the results and drafted the manuscript, EF designed the study, interpreted the results and drafted the manuscript, DBP assisted in the statistical analysis, CP critically reviewed the manuscript, CS critically reviewed the manuscript, SA interpreted the results, designed the study and critically reviewed the manuscript. All authors read and approved the final manuscript.
